# Adherence to medication in neurogeriatric patients: an observational cross-sectional study

**DOI:** 10.1186/s12889-019-7353-5

**Published:** 2019-07-29

**Authors:** Tino Prell

**Affiliations:** 1 Department of Neurology, Jena University Hospital, Am Klinikum 1, 07747 Jena, Germany; 2 Center for Healthy Ageing, Jena University Hospital, Jena, Germany

**Keywords:** Nonadherence, Stroke, Parkinson’s disease, Elderly, Health-related quality of life

## Abstract

**Background:**

Poor adherence is a major issue and is associated with increased morbidity, mortality, and immense costs for the healthcare system. Due to demographic changes, the burden of neurological diseases is increasing with a crucial exacerbation of the problem of nonadherence. However, comprehensive data on geriatric patients with neurological disorders do not exist to date. In this cross-sectional observational study we aim to identify disease-specific adherence-modulating factors in neurogeriatric patients.

**Methods:**

Patients 60 years or older with neurological disorders will receive an assessment of adherence (Stendal Adherence with Medication Score) and a comprehensive geriatric assessment during their stay in the Department of Neurology or Geriatrics at the Jena University Hospital (baseline data). In addition disease specific data will be derived from medical records. After one and twelve months a telephone interview will be conducted to evaluate if and why changes of medication occurred (follow up data).

**Discussion:**

This study aims to explore disease-specific patterns of nonadherence in elderly patients with neurological disorders and characteristics of information transfer between a specialized center, practicing neurologists, general practitioners, and the patients and their caregivers. This comprehensive data may help to develop and apply complex and disease-specific interventions to enhance adherence.

**Trial registration:**

German Clinical Trials Register DRKS00016774. Registered 19.02.2019.

## Background

The treatment of chronic disorders commonly includes the long-term use of pharmacotherapy and non-pharmacological therapy. However, their full benefits are often not realized because approximately up to 50% of patients either do not take medications as prescribed or do not follow recommendations [1]. In the geriatric population, nonadherence contributes to adverse drug events, increased length of stay and readmissions to hospitals, and a lower quality of life [2, 3]. However, physicians often do not routinely enquire about, and are therefore unaware of the extent of patients´ nonadherence to medication. Factors contributing to nonadherence are numerous [4]. Nonadherence is a dynamic process and may be intentional (when the patient purposefully decides not to follow the recommended treatment) or unintentional (when the patient cannot follow the recommendation) [5]. Not fully understood is why nonadherence often occurs after discharge from hospital. This is probably due to lack of care and routine that was available during patient’s stay in hospital. In Germany, the gap in medical care in hospitals compared to outpatient care is considerable and contributes both to nonadherence and to the frequently observed modifications in the medication regimen after discharge from hospital [6]. This process is influenced by several players and modifiers, such as poor communication, lack of intrinsic involvement of patients, lack of confidence in the physician’s professionalism, and different reimbursement systems. In particular, poor communication between different players in medical care, and feedback from practitioners to the hospital has to date not been sufficiently studied in neurogeriatric patients. This poor communication process may have a strong impact on adherence and patient-reported outcome.

Because the reasons for nonadherence are complex and diverse, interventions to improve adherence must be multifactorial. Although many interventions for increasing adherence have been tested, the best methods to improve adherence still remain unclear [7]. Moreover, the available data are mostly restricted to internal medicine. However, it seems reasonable to assume that the effectiveness of intervention strategies may be disease-specific. Surprisingly, the characteristics of nonadherence in patients with predominant neurogeriatric problems have not been sufficiently characterized, although such disorders – weakness, numbness, poor balance, stroke, Parkinson’s disease, and seizures – become more common with age. Over 20% of adults aged 60 and older suffer from a mental or neurological disorder (excluding headache disorders) and 6.6% of all disability (disability-adjusted life years-DALYs) among adults over 60 is attributed to neurological and mental disorders (WHO Fact sheet 04/2016).

While there are multiple systematic reviews concerning nonadherence in internal medicine (hypertension, COPD, asthma, HIV etc.), little is known about mechanisms of nonadherence in neurogeriatrics [7, 8]. Hence, we aim to collect comprehensive data for adherence and its modifying factors in geriatric patients with neurological disorders. Because drug adherence is a dynamic process, we aim to study a yet underrepresented modifier of adherence: the process after discharge from hospital and attending the outpatient clinic. Our comprehensive data can be used to develop and apply complex and disease-specific interventions to enhance adherence. In parallel we will evaluate the mechanisms of information flow between hospital, practitioner, patient, and caregivers and its impact on adherence and patient-reported outcomes. Therefore, the project will address the following questions:

### Research questions

- What are the disease-specific differences and predictors of adherence in neurogeriatric patients?
- How does the process after patient’s discharge from hospital influence adherence? What are the characteristics of information transfer between a specialized center (university hospital), practicing neurologists, general practitioners, and the patient and their caregivers? What are barriers and facilitators?

### Hypothesis

We hypothesize that nonadherence is also influenced by the underlying neurological disease and that results from patients with arterial hypertension cannot simply be translated one-to-one to neurological disorders. It also seems reasonable that different diseases, such as Parkinson’s disease or epilepsy follow different patterns and predictors for nonadherence. Therefore disease-specific interventions may be more helpful than a general intervention.

## Methods/design

### Setting and participants

Patients 60 years or older with neurological disorders will receive an assessment of adherence, a comprehensive geriatric assessment during their stay in the Department of Neurology or Geriatrics at the Jena University Hospital. Moreover, general data, prescribed medication regime, and disease-specific data about the neurological disorder will be extracted from medical records (baseline data). After one and twelve months a telephone interview will be conducted to evaluate post-discharge changes of medication (follow up data). The planned period of recruitment is from February 2019 to February 2020. The data collection for follow up interviews will take until February 2021.

Inclusion criteria: geriatric patients (defined as age > 60 with multimorbidity OR age > 70) with a common neurological disorder (cerebrovascular disorders, movement disorders, epilepsy, neuromuscular / peripheral neurological disorders).

Exclusion criteria: dementia, acute psychotic symptoms, delirium.

To avoid selection bias all geriatric patients in the Department of Neurology will be screened for eligibility. Known predictors of nonadherence (e.g. depression) will be assessed in detail to avoid bias in the analysis (Table 1).

**Table 1:** Specific assessments at baseline

Adherence	Stendal Adherence with Medication Score (SAMS)	18-items addressing reasons for nonadherence
Emotion, Depression	Beck Depression inventory II (BDI)	21-question multiple-choice inventory, for measuring the severity of depression
Mobility - Risk for falls	Timed “Up & Go“	Simple test to assess a person’s mobility and risk of falling
Cognition	Montreal Cognitive Assessment (MoCA)	30-point test assessing several cognitive domains with high sensitivity and specificity for detecting mild cognitive impairment
Personality	Big-Five-Inventory-10 (BFI-10)	10-item scale measuring the Big Five personality traits Extraversion, Agreeableness, Conscientiousness, Emotional Stability, and Openness
Patient physician relationship	Health Care Climate Questionnaire (HCCQ)	Measures outpatients’ experience of communication with physicians
Health-related quality of life	Short Form (36) Health Survey (SF-36)	Widely used 36-item measure of health status. It consists of eight scaled scores, which are the weighted sums of the questions in their section. The lower the score the more disability. The results can be summarized in two main composite scores: the physical composite score (PCS) and the mental composite score (MCS)

### Endpoints

The primary outcome is nonadherence according to the Stendal Adherence with Medication Score (SAMS) [5, 9]. We will determine disease-specific predictors of nonadherence in neurogeriatric patients taking personal, environmental and procedural factors into consideration.

### Sample size

The aim is to estimate nonadherence in common neurogeriatric patient subgroups (cerebrovascular disorders, movement disorders, epilepsy, neuromuscular / peripheral neurological disorders). A summary score measuring adherence as percentage is derived from the SAMS as described before [5]. Null percentage is defined as complete adherence. We expect significant nonadherence in about 25% and moderate nonadherence in 50% of the patients. Sample size calculation is based on the precision of our estimate for single proportions using nQuery Advisor 7.0 at 95% confidence interval. When the sample size of one patient subgroup is 250, a two-sided 95% confidence interval for a single proportion using the large sample normal approximation will increase by 5.4% from the observed proportion for an expected proportion of 25% of non-adherent patients.

### Recruitment of patients

Eligible participants are continuously recruited by trained study staff during their stay on the ward or during a visit in a specialized outpatient hospital and center (Departments of Geriatrics and Neurology from the Jena University Hospital, Jena, Germany). Because there are no harmful interventions planned, the willingness of patients and caregivers to take part in the study will be high.

### Data collection

#### Baseline

Data will be collected by trained study staff by applying specific assessments (Table 1) and data will be derived from the routine medical records. The data from the medical records include: general clinical and socio-demographic information, medication regimen, over-the-counter medications, specific information related to neurological disorder: specific disease-severity scales (e.g., Unified Parkinson’s Disease Rating Scale, National Institutes of Health Stroke Scale etc.). Self-administered scores by the patients will be immediately checked for completeness.

Follow up:

Semi-structured follow up telephone interviews with the patients or caregivers will be undertaken one and 12 months after discharge from hospital (up to three attempts to reach the patients). Here, we will ask if there was any change of prescribed medication, if yes why and who undertook the changes of medications. Moreover, the SF-12 and survival rate will be assessed after 12 months. We decided to use a detailed health-related quality of life questionnaire (SF-36) at baseline to better characterize the cohort and a shorter version for the telephone follow up interview because the long version is not practicable in addition to the interview. Both scores (SF-36 and SF12) show a high concordance with each other.

All data will be recorded using pseudonyms in a Windows Access Database.

#### Statistical analysis

Descriptive statistics will be used to describe i) data of the overall study population, ii) clusters of nonadherence, and iii) reasons for longterm changes of prescribed medication. Subjects with missing baseline data will be excluded from analysis. Non-participation will be divided into loss to follow-up (i.e., failure to locate the individual, death) and non-response to the follow-up survey. An exploratory generalized mixed model with random effects (time-point) will be applied to identify possible explanatory factors for nonadherence. We will use nonadherence (yes/no, depending on the SAMS) as dependent variable [5]. Explanatory variables will be patient group (different neurological diseases), age, gender, marital status, education, functional and motor performance (disease specific scales), depression (BDI), and cognition (MoCA), and physician patient relationship (HCCQ). We generally apply a significance level of 0.05 and 2-sided tests.

## Discussion

Nonadherence is a major issue in health care and the current study aims to improve our understanding of nonadherence in elderly patients with neurological disorders. For this purpose we will analyze the relationship between adherence and social, clinical and environmental parameters. Moreover, we take into account that adherence is a dynamic process and that different players in different health care settings may influence longterm adherence in elderly patients.

The study is not free of limitations. In this monocentric study we will mainly address personal factors contributing to nonadherence, because we are interested in individual reasons of nonadherence. We do not apply other techniques to measure adherence (e.g. electronic pill count) to determine the degree of adherence. Therefore the known restrictions of self-report adherence measures have to be taken into account [10, 11]. Nevertheless, our comprehensive data can be used to develop and apply complex and disease-specific interventions to enhance adherence.

### Trial status

Data collection has started on 19.02.2019 and is currently ongoing.

## Data Availability

The datasets generated and/or analysed during the current study will be available from the corresponding author on reasonable request.

## Abbreviations

BDI: Beck Depression inventory II
BFI-10: Big-Five-Inventory-10
HCCQ: Health Care Climate Questionnaire
MoCA: Montreal Cognitive Assessment
SAMS: Stendal Adherence with Medication Score
SF-12: Short Form (12) Health Survey
SF-36: Short Form (36) Health Survey
